# Fracture of a Femoral Revision Stem following a Technical Failure

**DOI:** 10.1155/2018/9691627

**Published:** 2018-06-19

**Authors:** Falko Herold, Henk Eijer

**Affiliations:** Department of Orthopaedic Surgery, RS Emmental AG, Burgdorf, Switzerland

## Abstract

We report about a fracture of a modular, uncemented femoral revision stem (Zimmer, Revitan®) due to a not previously described intraoperative technical problem. During implantation, a small ring, part of the proximal part of the trial stem, was left on the distal part of the definite stem. Following this, the top screwcap of the proximal part of the definite stem could not be tightened properly. However, the stem was thought to be stable, and the situation left. Two and a half years later, the proximal part of the stem fractured and the situation became unstable. It is very useful to know about this ring and that it should always be removed, otherwise, there is possibility that it may be left on the definite distal part of the stem with the possibility of a later fracture.

## 1. Introduction

Fracture of a stem of a modular hip revision arthroplasty is not an entirely uncommon complication. As many other types of revision hip stems, the Zimmer Revitan stem is a modular shaft, which provides the surgeon many options for restoring leg length, hip offset, and femoral anteversion and therefore good stability. In general, and also with this stem, it is thought that the advantages of the modularity of the stem outweigh its susceptibility to corrosion, fretting, and fatigue fracture [1–4]. Several reports have been published about fractures of a modular femoral stem. Nearly always, the fracture is seen in the middle of the two parts of the stem. Although failure may be due to the morphological construction of the stem, in many cases noningrowth of the proximal part of the femur to the proximal part of the stem is thought to be responsible for failure.

We describe a failure of a Revitan revision stem following an intraoperative problem.

## 2. Case Description

**Figure 1:**
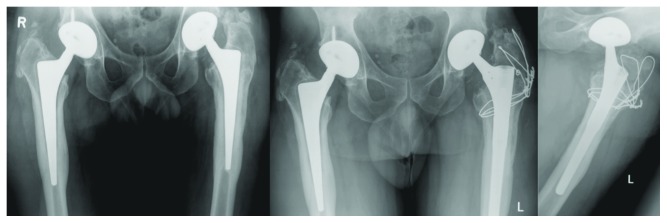
Radiograph a.p. view with loosening of the left femoral stem, radiograph a.p. and axial view of first revision.

In March 2008, a primary total hip replacement was revised for loosening of the femoral stem in a 48-year-old carpenter (Figure 1). The old lateral incision with an approach including an osteotomy of the greater trochanter was used. A Zimmer Revitan stem (distal part: diameter 14 mm, length 140 mm; proximal part: length 75 mm, conical, taper 12/14; head Biolox diameter 28 mm). The osteotomy was fixed with cerclage wires intraoperatively. The definite distal part was implanted first and tested with a trail proximal part. Then, the surgeon experienced difficulties with the connection of the definite proximal part of the stem to the definite distal part. Although it fitted, the screw to fix the two parts together could not be tightened. The surgeon even contacted the company during the operation but failed to find an explanation for the problem. As the 2 parts seemed to be fixed well together, he left the situation as it was. The postoperative radiographs (Figure 1) were acceptable, and no explanation for the intraoperative problem was found. The patient did well, and there were no complications.

**Figure 2:**
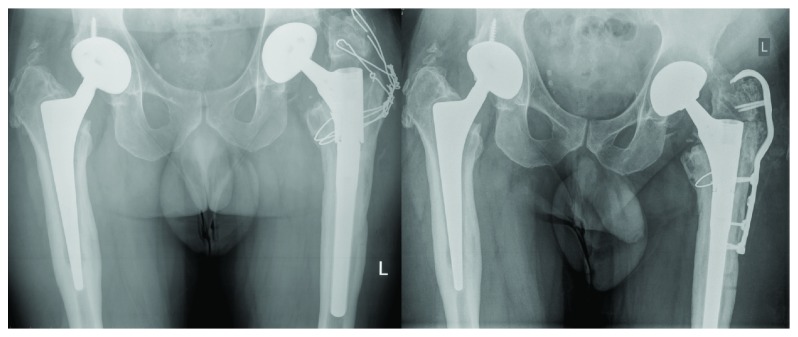
Radiograph a.p. view with broken stem and radiograph a.p. view of the second revision.

Two and a half years postoperatively, the patient felt a sudden pain while walking. He could only limp and hardly walk. He walked on without consulting a doctor for another 2 months, where after he presented to us. On the radiographs, a fracture in between the 2 modular parts of the stem was seen (Figure 2).

**Figure 3:**
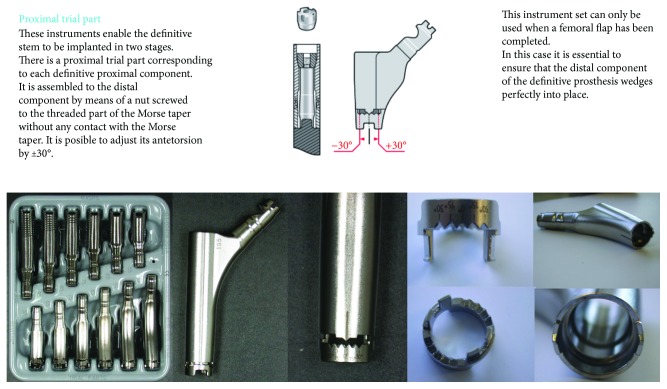
Description of the manufacturer's operation manual [5]; small ring, part of the proximal part of the trail stem.

A rerevision was performed using the same approach and through the nonunion of the greater trochanter. Intraoperatively, the proximal part of the stem was found broken on the medial side. The distal part was well fixed, and there seemed to be no need to change this part. Even the threaded part of the distal stem was intact. However, connection of a new proximal part to the distal part failed, as during the first revision. On further inspection, a small ring was suddenly born from the distal part. It was then discovered to be a part of the proximal trial part (Figure 3). Thereafter, connecting the two parts was easy. The nonunion of the trochanter was debrided and fixed with a hooked plate (proximal conical spout Revitan 75 mm, Biolox Delta Ceramic femoral head 28 mm medium, Trofix Trochanter Fixation plate large head 150 mm) (Figure 2).

Postoperatively, the patient did well, and 5 years after the second revision, the patient is still without pain, hardly, and back to work.

## 3. Conclusions

Fracture of a stem of a modular hip revision arthroplasty is not an entirely uncommon complication. As many other types of revision hip stems, the Zimmer Revitan stem is a modular shaft, which provides the surgeon many options for restoring leg length, hip offset, and femoral anteversion and therefore good stability [5]. In general, and also with this stem, it is thought that the advantages of the modularity of the stem outweigh its susceptibility to corrosion, fretting, and fatigue fracture [1–4]. Several reports have been published about fractures of a modular femoral stem. Nearly always, the fracture is seen in the middle of the two parts of the stem [1, 2]. Although failure may be due to the morphological construction of the stem, noningrowth of the proximal part of the femur to the proximal part of the stem is thought to be responsible for failure [4]. This is the first report of a fracture of the proximal part of the uncemented Zimmer Revitan revision stem caused by unrecognized loosening of the distal ring of the proximal trial part, which results in a stem fracture and disassociation (Figure 3).

In our patient, the fracture did not occur in between the two parts. There was a fracture of the proximal part of the stem following disassociation of the two parts that were never fixed together well. We were lucky to be able to use the distal part of the stem again, as it was still well fixed to the bone. It seems to us that this complication is due to the design of the trial prosthesis, although of course the surgeon is always responsible for proper handling of the instruments and trial implants. However, we call into question the necessity this ring on the proximal part, this particular trail part. One should always be sure that this ring is removed before assembling the complete prosthesis in vivo.
